# Post Deployment Care for Returning Combat Veterans

**DOI:** 10.1007/s11606-012-2061-1

**Published:** 2012-05-31

**Authors:** Juliette F. Spelman, Stephen C. Hunt, Karen H. Seal, A. Lucile Burgo-Black

**Affiliations:** 1 VA Connecticut Health Care System, 950 Campbell Ave 11 ACSL, West Haven, CT 06511 USA; 2 Yale School of Medicine, West Haven, CT USA; 3 Puget Sound VA Medical Center, Seattle, WA USA; 4 University of Washington School of Medicine, Seattle, WA USA; 5 San Francisco VA Medical Center, San Francisco, CA USA; 6 Departments of Medicine and Psychiatry, University of California, San Francisco, San Francisco, CA USA

**Keywords:** combat, post-deployment, OEF/OIF, veteran, Iraq, Afghanistan

## Abstract

Since September 11, 2001, 2.4 million military personnel have deployed to Iraq and Afghanistan. To date, roughly 1.44 million have separated from the military and approximately 772,000 of these veterans have used VA health care. Combat deployments impact the physical, psychological, and social health of veterans. Given that many veterans are receiving care from non-VA providers, it is important that all community health care workers be familiar with the unique health care needs of this patient population, which include injuries associated with blast exposures (including mild traumatic brain injury), as well as a variety of mental health conditions, such as post-traumatic stress disorder. Other important health concerns are chronic musculoskeletal pain, medically unexplained symptoms, sequelae of environmental exposures, depression, suicide, substance abuse, sleep disturbances, and impairments in family, occupational and social functioning. Elevated rates of hypertension and tobacco use remind us that deployment may result not only in immediate impacts on health, but also increase risk for chronic disease, contributing to a growing public health burden. This paper provides a comprehensive review of these health concerns and offers practical management guidelines for primary care providers. In light of relationships between physical, psychological and psychosocial concerns in this population, we recommend an interdisciplinary approach to care directed toward mitigating the long-term health impacts of combat.

A 24-year-old combat veteran presents to your clinic. He returned from Iraq 5 months ago after a year-long deployment as an army medic. He was exposed to several blasts from improvised explosive devices, and tells you he suffered a traumatic brain injury after one incident during which he momentarily blacked out. He injured his back running on uneven ground while wearing body armor, and has chronic pain for which he is taking oxycodone. He reports having a “short fuse,” and feels “constantly on edge”; he also reports difficulty sleeping and frequent vivid nightmares. He drinks at least six beers a night to calm down. His wife is upset that he doesn’t trust anyone, and she is frightened that he sleeps with a gun under his pillow. He has been unable to find a steady job, citing an inability to concentrate at work. He tells you “it has gotten to the point where I really don’t care what happens to me…”

As illustrated in this case, deployment to a war zone is an experience that may profoundly impact a veteran’s health and personal life, carrying the risk of long-term physical, psychological, and social impairments. Effectively managing these global health concerns can be a daunting task. Our objective is to orient primary care providers to the specific health concerns of this unique patient population, and to offer practical management tools to facilitate care. This narrative review is divided into two main sections. The first provides a detailed summary of the multiple health risks imposed by deployment and combat. The second offers evidence-based practical management suggestions for the most common of these concerns.

## EPIDEMIOLOGY

Approximately 2.4 million US military personnel have served in Afghanistan and Iraq in Operation Enduring Freedom (OEF), Operation Iraqi Freedom (OIF), and Operation New Dawn (OND) since the beginning of these conflicts in 2001. To date, roughly 1.44 million individuals have separated from the military and are eligible for VA services and about 772,000 have used VA health care^1^ (Table 1).

**Table 1:** Demographic Characteristics of OEF/OIF/OND Veterans Utilizing VA Healthcare (*N* = 771,874)

Characteristics	Percent (%)
Sex	
Male	88.0
Female	12.0
Age	
17–31	46.8
32–41	25.7
42–51	20.5
52–61	6.0
62–85	0.9

Table adapted from VA cumulative data from 1st Quarter FY 2002 through 1st Quarter FY 2012

The three most prevalent conditions seen in recent combat veterans are musculoskeletal problems, mental health conditions and "non-specific signs and symptoms"^1^ (Table 2). Commonly referred to as “medically unexplained symptoms,” this third category may include fatigue, somatic, and cognitive complaints (memory, attention and concentration difficulties).^2^–^4^

**Table 2:** Frequency of ICD9 Diagnosis in OEF/OIF/OND Veterans Utilizing VA Healthcare (*N* = 771,874)

Diagnosis (Broad ICD-9 Categories)	Frequency (Percent)
Diseases of Musculoskeletal System/Connective System (710–739)	434,552 (56.3)
Mental Disorders (290–319)	404,060 (52.3)
Symptoms, Signs and Ill Defined Conditions (780–799)	396,592 (51.4)
Diseases of Nervous System/Sense Organs (*includes hearing loss and tinnitus) (320–389)	342,161 (44.3)
Disease of Digestive System (*includes Dental concerns) (520–579)	276,122 (35.8)
Diseases of Endocrine/Nutritional/ Metabolic Systems (240–279)	245,215 (31.8)
Injury/Poisonings (800–999)	221,242 (28.7)
Disease of Respiratory System (460–519)	200,201 (25.9)
Diseases of Skin (680–709)	163,973 (21.2)
Diseases of Circulatory System (390–459)	162,921 (21.1)
Infectious and Parasitic Diseases (001–139)	118,860 (15.4)
Diseases of Genitourinary System (580–629)	114,982 (14.9)
Benign Neoplasms (210–239)	50,406 (6.5)
Diseases of Blood and Blood Forming Organs (280–289)	28,495 (3.7)
Malignant Neoplasms (140–209)	10,498 (1.4)

## RISKS OF COMBAT AND DEPLOYMENT

### Combat Injury

Recent improvement in personal protective gear and battlefield emergency medical care has resulted in decreased mortality, yet increased morbidity in soldiers surviving their injuries.^5^ Currently, many surviving soldiers face serious long-term consequences such as amputations, spinal cord injuries, and traumatic brain injuries. The most common cause of injury among these veterans is blast waves from improvised explosive devices (IEDs) or rocket propelled grenades (RPGs).^6^,^7^ Traumatic brain injury (TBI) is often referred to as the “signature injury” of the current conflicts.^8^ Depending on the study and definition, the incidence of mild to moderate TBI has been reported to be as high as 10–20 %.^8^–^11^ The patient described above experienced multiple IED blasts and is concerned that he has a brain injury. His problem concentrating and inability to stay employed may stem from a TBI, but may also be related to possible posttraumatic stress disorder, alcohol abuse, sleep disturbances, current psychosocial stressors and opiate misuse, or, as is most often the case, a combination of these factors.

### Occupational Exposures

Other physical risks inherent in the combat environment include exposure to austere living conditions and harsh climate. In Iraq and Afghanistan daily temperature extremes range from over 100 degrees Fahrenheit during the day to subfreezing temperatures at night. There are frequent sand storms and hygiene may be severely compromised. Heat stroke, frostbite, upper and lower respiratory complaints, fungal or bacterial skin infections, and dental concerns are common. High impact noise from weapons, vehicles, and aircraft can result in acoustic injury; hearing loss and tinnitus are among the most common service connected health conditions.^12^ Military personnel often carry heavy combat equipment, including body armor, weaponry, and supply packs, sometimes exceeding 100 pounds. Men and women are issued the same equipment, often with little accommodation for body size. Women have served in combat zones, and many are exposed to the same combat risks as their male counterparts.^13^ Urinary infections are common in women, a consequence of dehydration and delayed micturition due to inadequate privacy.

Many returning veterans also report hazardous environmental exposures, the long-term health effects of which may remain unknown for many years. Smoke from burn pits where garbage and human waste are incinerated are the most commonly reported, followed by exposure to sand storms. A small, recent descriptive case series of veterans with persistent unexplained dyspnea after deployment in Iraq and Afghanistan suggests a possible association between diffuse constrictive bronchiolitis and inhalational exposures.^14^ In addition, those serving may be exposed to infectious agents, such as malaria, tuberculosis, diarrheal illness from amoebiasis or giardiasis, and skin or systemic infections from leishmaniasis.^15^

### Deployment/Combat Related Chronic Health Problems

The Millennium Cohort Study is a joint Veterans’ Affairs (VA)–Department of Defense (DoD) prospective longitudinal study that examines the health impacts of war. At baseline, 77,047 US service members were surveyed between 2001 and 2003. Of note, the pre-deployment baseline functional health status of this cohort was superior to that of the general population of the same age and sex distribution.^16^ Millennium Cohort data suggest that deployment is associated with higher rates of smoking initiation and smoking recidivism than age- and gender-matched civilians, particularly with prolonged deployment, multiple deployments, or combat exposure.^17^ The prevalence of smoking in younger veterans is as high as 40 % (compared with 20 % in non veterans).^18^ Combat has been linked to other cardiovascular risk factors, including hypertension; deployed individuals reporting multiple combat exposures were 1.33 times more likely to be diagnosed with hypertension compared with individuals who deployed, but did not en*g*age in combat.^19^ A large retrospective analysis of cardiovascular risk factors in OEF/OIF/OND veterans with co-morbid mental health diagnoses found elevated rates of reversible cardiovascular risk factors, including tobacco use, hypertension, dyslipidemia, obesity, and diabetes.^20^ Another cross sectional study demonstrated a greater burden of medical illness in OEF/OIF/OND men and women VA users with co-morbid post-traumatic stress disorder (PTSD) compared with those without mental health problems.^21^ These data are particularly concerning given the high incidence of PTSD and other mental health disorders in this population.

Chronic pain is prevalent in the returning veteran population. In one study, approximately 47 % reported mild levels of pain, and 28 % reported moderate to severe pain.^22^ In a more recent smaller retrospective review of 429 veterans seen at a specialized post-deployment center, 29 % of patients screened positive for chronic widespread pain. When present, chronic pain was related to poorer physical function.^23^ In a small sample of patients referred to a poly-trauma network site, chronic pain was found to be frequently co-morbid with both PTSD and persistent post-concussive symptoms.^24^ In summary, while OEF/OIF/OND personnel have better overall physical health at baseline than the general population, deployment and combat both have significant immediate impacts on important health outcomes including injury, medical illness, and increased chronic disease risk factors, that may well persist over a lifetime and contribute to a growing public health burden.

## MENTAL HEALTH RISKS

There are many psychological stressors associated with combat deployment. These include anticipation of combat, combat and noncombat-related psychological trauma, military sexual trauma, and separation from home and family. Rates of post-deployment mental health conditions are high. A recent study of approximately 289,000 veterans with ICD-9 coded mental health diagnoses estimated a prevalence of 36.9 % for mental health diagnoses, with 21.8 % diagnosed with PTSD and 17.4 % diagnosed with depression.^25^ These percentages are considerably higher than earlier reports;^26^–^28^ whether this reflects more effective mental health screens, or an actual increased burden of disease over time remains unclear. Data support higher rates of depression for women veterans compared to their male counterparts. Women also more commonly report military sexual trauma,^29^ and are at increased risk of disordered eating and weight loss.^30^ An increased risk of new onset heavy weekly drinking, binge drinking, and alcohol-related problems has been reported in younger deployed service members exposed to combat versus noncombat-exposed personnel.^31^ There are also high rates of substance use disorders,^32^ and other anxiety disorders. “Sub-syndromal” mental health symptoms such as sleep disturbances and irritability may also cause significant distress without meeting criteria for any specific diagnosis.^33^ Rates of suicide in younger male veterans are elevated compared to civilians.^34^

## PSYCHOSOCIAL RISKS

Psychosocial risks of combat deployment include marital instability, unemployment/ underemployment, financial decline, social isolation, and legal problems, as reported in Vietnam-era veterans.^35^ In addition, recent evidence suggests a higher incidence of mental health diagnoses in spouses of veterans who have deployed, particularly if there has been a prolonged deployment.^36^ Moreover, mental health conditions negatively impact psychosocial functioning. The resultant deterioration of social networks may lead to increasing mental health symptoms. This dangerous cycle of decline can only be interrupted by providing effective mental health treatment and by helping veterans preserve and strengthen support networks. The broad impacts of combat deployment on marital and family health and overall functioning have been summarized in a recent Institute of Medicine report.^34^ Among other recommendations, the report calls for additional research to study the impacts of combat deployment on families and evaluate existing "support programs to maximize their reach and effectiveness."

## MANAGING COMMON HEALTH CONCERNS OF RETURNING COMBAT VETERANS

### Musculoskeletal Pain

Musculoskeletal injury with chronic pain is the most common health concern in this patient population. Pain relief needs to be approached from a functional, rehabilitative perspective utilizing pharmacologic, behavioral, and alternative treatment modalities. These include physical therapy, massage, TENS, thermal and aqua therapy, encouragement of regular exercise, chiropractic treatment, and acupuncture. Incorporating behavioral strategies into a comprehensive, collaborative treatment plan involving a health psychologist for cognitive behavioral therapy, biofeedback training, stress management, and alternative techniques such as deep relaxation training, meditation, and yoga have been shown to improve pain outcomes.^37^,^38^ Co-morbid mental health conditions or psychosocial stressors may lower the pain threshold and augment pain experiences.^39^ Thus, addressing mental health concerns and providing adequate social work/case management support with attention directed to family issues and vocational support can be very helpful.

Opiates should be used with caution and reserved for refractory chronic pain conditions, given the high risk for abuse. Additionally, co-morbid mental health conditions compound the risk for inappropriate use of opiates, as veterans may attempt to self-medicate their "psychological pain".^40^ PTSD increases risk for opiate prescription, high risk opiate use and adverse events.^41^ If a combat veteran is already on opiates, a careful re-assessment of therapeutic need is warranted. It may be appropriate to shift from opiates to more sustainable long-term interdisciplinary pain management strategies. In the case above, we would recommend slowly transitioning off opiates to alternative analgesics, referral to a chiropractor or physical therapist, and further mental/behavioral health assessment and treatment.

### Sleep

Sleep disturbances are common, as the often unpredictable environment of the war zone can disturb diurnal cycles, even after veterans return home. Both sleep quality and quantity are negatively impacted by deployment.^42^ We encourage teaching basic sleep hygiene and referral to a health psychologist for cognitive behavioral therapy or motivational support for healthy lifestyle changes when available. For more severe insomnia, one might start with sedating antihistamines or trazodone. The use of benzodiazepines is strongly discouraged given risks for misuse, though partial benzodiazepine agonists (such as zolpidem) can be used, as indicated, in conjunction with sleep hygiene and cognitive behavioral approaches. In addition, prazosin, slowly titrated to higher doses (1–15 mg) has been shown to be effective for PTSD-related nightmares that disturb sleep.^43^

### Recognizing and Treating PTSD

Given the high rates of co-morbid PTSD and depression, clinicians should routinely assess for both. Because there is the potential for delayed onset and the rapid progression of symptoms, current VA/DoD guidelines recommend reassessment for PTSD and depression within 3 to 6 months and annually thereafter. It should be noted that those who screen negative for PTSD, but have sub-syndromal trauma-related symptoms may carry a high risk for suicide.^44^ If trauma related symptoms are present, a suicide risk assessment is appropriate given the increased risk for suicide in this population. The veteran described here expressed that he does not care what happens to him. This should raise concern for possible suicidality and prompt immediate assessment.

Primary care providers should recognize the hallmark signs and symptoms of PTSD. Exposure to a perceived life-threatening experience is necessary for diagnosis. PTSD is characterized by re-experiencing (such as intrusive thoughts or recollections, or nightmares), avoidance or numbing, and increased arousal (such as insomnia, irritability, or the classic startle response).^45^ Our patient has symptoms in all three symptom clusters: his re-experiencing takes the form of frequent vivid nightmares, his avoidance/numbing is in his lack of trust and use of alcohol, and he clearly is irritated and anxious (hyper-arousal symptoms). Nevertheless, clinicians should be cautious about diagnosing PTSD in the initial phases of readjustment. PTSD is highly co-morbid with other mental health problems, such as depression and panic disorder; establishing an accurate diagnosis can be complicated. Often, veterans do not wish to be labeled and feel that by seeking treatment they are admitting to weakness. Misperceptions about PTSD should be corrected and normalized by explaining that post-traumatic stress symptoms are quite common in the post-deployment period.

Referral for specialty PTSD treatment may be indicated if symptoms are severe or disabling. Specialized treatment for PTSD involves cognitive behavioral therapy (CBT), which has been empirically validated as the most effective treatment.^46^ CBT can include exposure therapy or cognitive processing therapy, both of which involve repeatedly re-visiting a single traumatic event in several extended sessions over several weeks. Other non-CBT approaches include stress and anger management, biofeedback, interpersonal therapy, and group therapy. Selective serotonin re-uptake inhibitors (SSRIs) have also demonstrated efficacy; sertraline and paroxetine are two FDA-approved medications for the treatment of PTSD. SSRIs have been found to be most effective in combination with therapies such as CBT.^47^

Though there can be clear and lasting benefits from CBT, veterans may be resistant to mental health referral or find it difficult to participate in time intensive therapy, often relying solely on their primary care providers. Indeed, specialty mental health services remain underutilized,^48^ stressing the importance of a basic treatment approach in primary care. In our case, the primary care clinician’s role is to establish a safe place where patients feel comfortable sharing their experiences. This can involve simple reflective statements like, “It sounds like you’ve been through a lot. Would you like to share what you’re going through?” Clinicians should de-stigmatize mental health care: “It sounds like you are having a difficult time readjusting to life at home. Many combat veterans experience this. How would you feel about seeing a stress specialist?” By understanding and explaining therapeutic options, we can assist veterans in engaging in treatment. Primary care clinicians can consider initiating pharmacotherapy using SSRIs. According to VA/DoD guidelines, either an SSRI or CBT may be considered as first line therapy.^49^ Benzodiazepines should be avoided, due to the lack of data supporting benefit, and potential for abuse. In addition, significant distress can be mitigated by managing associated sleep problems. Clinicians should also closely monitor symptoms for progression, particularly in the early months and years following return from deployment.

### Traumatic Brain Injury

Traumatic Brain Injury (TBI) is defined as the result of a blow or jolt to the head or a penetrating injury that disrupts brain functioning.^50^ This disruption occurs immediately following an injury and may include one of the following: a loss or decreased level of consciousness, a loss of memory immediately before or after injury, any alteration in mental state at the time of injury (confusion/disorientation), or a neurological deficit (weakness/balance/sensory changes).

Common causes of TBI include blasts, bullets, shrapnel fragments, falls, or motor vehicle accidents. TBI’s can range in severity from mild TBI (often used interchangeably with the term concussion) to severe TBI. Severity is determined at the time of injury. There are different guidelines for judging severity, including the VA/DoD clinical practice guidelines (included in our list of resources). The vast majority of TBIs sustained in theater are mild and most patients recover completely within 1–3 months. Symptoms usually fall into four major categories: physical symptoms (dizziness/vertigo/nausea/headaches), neurosensory symptoms (vision problems/light sensitivity/hearing loss/numbness), cognitive symptoms (poor concentration/forgetfulness/difficulty organizing/completing tasks), and psychiatric symptoms (insomnia/anxiety/irritability).

Most patients with a mild TBI or concussion require only supportive treatment and reassurance that symptoms typically resolve within several months. Patients should get adequate sleep, avoid activities that can lead to a second brain injury, avoid alcohol or other medications which have a sedative or stimulant affect (including caffeine/energy drinks), and be taught coping skills to assist with memory or irritability. Clinicians can consider subspecialty referral if there is a significant impact on daily activities, persistent neuropsychological deficits, psychosocial distress, or confusion regarding appropriate diagnosis. This can involve referral for formal neuropsychiatric testing, to rehabilitation specialists for functional evaluation and cognitive remediation, or to mental health clinicians for treatment. VA/DoD clinical practice guidelines provide more detailed recommendations for management and referral of TBI (Text Box 1).^51^

Text Box 1. TBI Symptom management*

**Figure:**
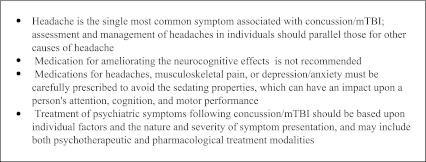

*adapted from VA/DoD Clinical Practice

Concussions and post-concussive symptoms are associated with an increased risk of PTSD, chronic pain and headaches;^52^,^53^ there is also significant overlap between post-concussive symptoms, chronic pain, and PTSD.^54^ Though it is often difficult, if not impossible, to attribute symptoms to a particular diagnosis, treatment is not dependent on this. A recent publication studying longitudinal effects of mild TBI and PTSD found little evidence for long term negative impact of mild TBI after adjusting for PTSD symptoms.^55^ Our patient described momentary loss of consciousness after a blast, suggesting that he sustained a concussion. His symptoms of irritability and lack of concentration could be manifestations of either the concussion or PTSD. In either case, limiting alcohol intake, appropriately managing opiate pain medications, and treating his anxiety, irritability, and insomnia, as well as addressing current stressors will be critical aspects of his care.

### Best Practices for Care

In light of the wide spectrum of post-combat health concerns, VA and DoD guidelines recommend an interdisciplinary approach involving integrated teams of primary care, mental health and social work providers which can normalize and de-stigmatize mental health treatment.^56^ Recognizing that co-located, interdisciplinary care may not be feasible for many providers, we suggest utilizing local resources and facilitating interagency collaboration with local Vet Centers or with the VA. Each VA facility has an OEF/OIF/OND program manager who coordinates patient care, provides case management, and accesses resources within the VA as well as in the local community. We have provided a list of available web based resources (Text Box 2).

**Text Box 2. Web Resources**

**Figure:**
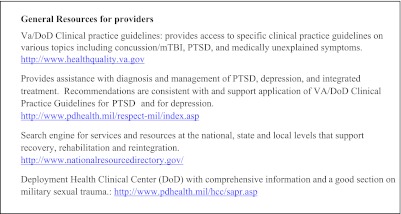

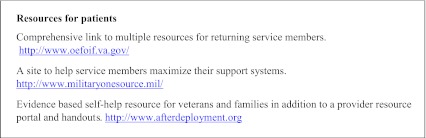

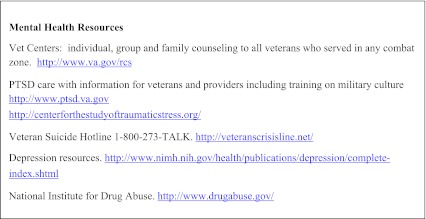

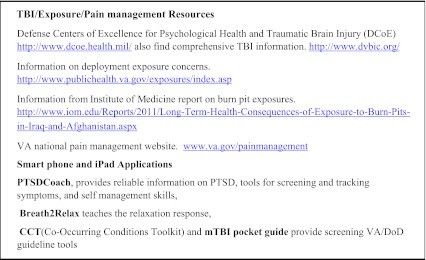

Based on the experiences of clinicians working in post-combat care programs in the VA, we have compiled a list of clinical "pearls" (Text Box 3). First, it is important to openly address barriers to care, particularly mental health care. Avoidance is a common coping strategy for those suffering from PTSD; depression may be characterized by low motivation and apathy; substance use is associated with denial and poor adherence; mild TBI is characterized by poor concentration and memory. All of these may interfere with successful engagement. Young families or competing work and/or school obligations may present additional barriers. Clinicians should acknowledge these barriers and offer support to overcome them. A welcoming, family-friendly environment can be very helpful. Other suggestions include expanding clinic hours to accommodate schedules, improving access and continuity, streamlining referrals, and arranging for shorter intervals between visits. A strong patient-provider relationship is essential in establishing therapeutic alliance and in encouraging the veteran to accept help. Expressing gratitude for the veterans’ service to their country and taking a brief military service history (Text Box 4) promotes this connection and contextualizes current health concerns in a way that the routine past medical history and social history may not. A review of systems focusing on common complaints may facilitate a symptom-based approach to care.

**Figure:**
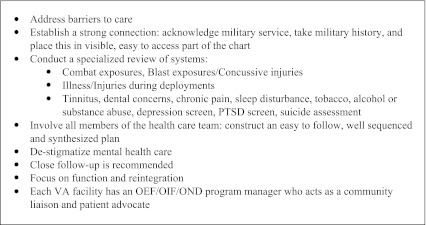

**Figure:**
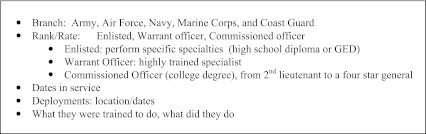

**Text Box 3. Clinical Pearls**

## CONCLUSION

In summary, our case illustrates that veterans returning from deployment to Iraq and Afghanistan are at risk for a wide variety of physical, psychological, and psychosocial health concerns. Often, there is significant overlap between the physical and psychological wounds of war; many patients have concurrent symptoms such as chronic pain, anxiety, insomnia, irritability, and cognitive disturbances. Primary care clinicians, in collaboration with mental health and social service providers, are well-positioned to play a central role in patient-centered, interdisciplinary, and integrated approaches to treatment. The complexity and breadth of health risks in this patient population raise concern for poor future health outcomes. Since many veterans are seeking health care outside the VA system, it is important that all providers have a familiarity with the health challenges faced by this patient population. The service and sacrifices of these veterans and their families require an effective community-wide system of care to support them as they recover and rejoin their communities.
